# Circadian Clocks, Stress, and Immunity

**DOI:** 10.3389/fendo.2016.00037

**Published:** 2016-05-02

**Authors:** Rebecca Dumbell, Olga Matveeva, Henrik Oster

**Affiliations:** ^1^Chronophysiology Group, Medical Department I, University of Lübeck, Lübeck, Germany

**Keywords:** circadian clock, HPA axis, immune system, glucocorticoids, stress

## Abstract

In mammals, molecular circadian clocks are present in most cells of the body, and this circadian network plays an important role in synchronizing physiological processes and behaviors to the appropriate time of day. The hypothalamic–pituitary–adrenal endocrine axis regulates the response to acute and chronic stress, acting through its final effectors – glucocorticoids – released from the adrenal cortex. Glucocorticoid secretion, characterized by its circadian rhythm, has an important role in synchronizing peripheral clocks and rhythms downstream of the master circadian pacemaker in the suprachiasmatic nucleus. Finally, glucocorticoids are powerfully anti-inflammatory, and recent work has implicated the circadian clock in various aspects and cells of the immune system, suggesting a tight interplay of stress and circadian systems in the regulation of immunity. This mini-review summarizes our current understanding of the role of the circadian clock network in both the HPA axis and the immune system, and discusses their interactions.

## Introduction

Life on Earth has evolved in the context of a rhythmic environment, characterized largely by the regular succession of night and day. This has led to the evolution of intrinsic circadian (from Latin *circa diem* – about the day) clock systems, in order to optimally time physiological and behavioral processes. Disruption of circadian timing, such as with inter-time zone travel, shift work, and mistimed eating, can have consequences for cardiovascular, metabolic, and mental health and, crucially, immune function. The hypothalamic–pituitary–adrenal (HPA) axis and the immune system show extensive crosstalk, in particular with regard to the strong anti-inflammatory effects of glucocorticoids (cortisol in humans and corticosterone in rodents). However, less well studied is the interaction of the HPA axis and immune system with regard to the circadian clock. This mini-review will summarize current knowledge regarding the role of the circadian clock in each of these systems, and the interactions that can occur in the context of disrupted circadian rhythmicity.

## The Circadian Clock

Circadian rhythms are synchronized to external time by cues known as zeitgebers (German for time givers), such as light and food. In mammals, the clock system is organized in a hierarchical manner, with a master pacemaker residing in the hypothalamic suprachiasmatic nuclei (SCN), acting to synchronize peripheral clocks in all other tissues *via* endocrine and autonomic signals (1). At the cellular level, circadian clocks coordinate gene expression programs to control physiological processes over the course of the day. The primary zeitgeber for the SCN is light. From the eye, signals are relayed *via* the retinohypothalamic tract to the SCN, which in turn coordinates peripheral tissue clocks. Other zeitgebers can influence circadian rhythms, with mistimed feeding in particular being able to reset peripheral clocks independent from the SCN (2, 3).

**Figure 1:**
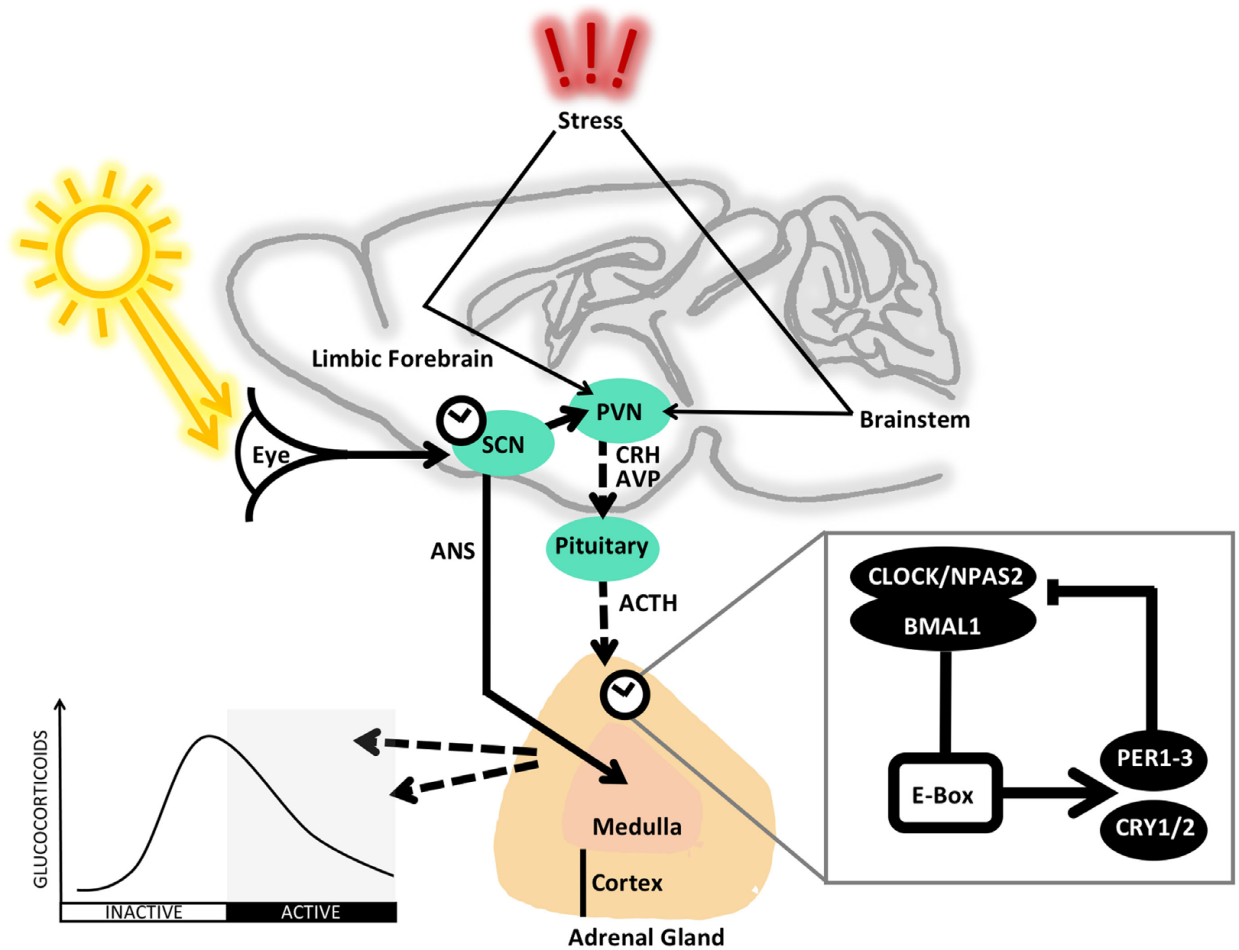
**The rhythmic control of the HPA axis is regulated at several levels**. The master clock residing in the suprachiasmatic nucleus (SCN) is synchronized by light information received *via* the retinohypothalamic tract from the eye in order to exert autonomic (ANS) and hormonal influence on the clocks and rhythms of downstream tissues of the body. In addition to the direct innervation of the adrenal, the SCN influences the paraventricular nucleus (PVN) to secrete corticotropin-releasing hormone (CRH) and arginine vasopressin (AVP), which reach the pituitary *via* the blood portal system to stimulate secretion of adrenocorticotropic hormone (ACTH), which activates production and release of glucocorticoids. In addition, local adrenal clocks are thought to regulate responsiveness to ACTH in a circadian fashion. The baseline circadian rhythm of circulating glucocorticoids peaks just before the beginning of the active phase (day in humans and night in rodents). Stress-induced stimulation of the HPA axis acts *via* afferent signals from the limbic forebrain and brainstem to the PVN. Inset: the core transcriptional–translational feedback loop (TTL) that makes up the molecular circadian clockwork. In the positive arm of the clock, CLOCK or NPAS2 form a complex with BMAL1 and bind to *E-Box* elements in the gene promotors of PERs and CRYs, which make up the negative arm and act to inhibit the activity of CLOCK–BMAL1 or NPAS2–BMAL1, with a cycle of roughly 24 h. For further detail, see the main text.

The molecular circadian clock consists of interlocked transcriptional–translational feedback loops [TTLs; discussed in detail elsewhere (4, 5)]. Briefly, during the day, the transcription factors CLOCK (circadian locomotor output cycles kaput) or NPAS2 (neuronal PAS domain-containing protein 2) in complex with BMAL1 (brain and muscle aryl hydrocarbon receptor nuclear translocator-like 1) bind to *E*-*box* (enhancer box) promoter elements to drive expression of *Period* (*Per1-3*) and *Cryptochrome* (*Cry1/2*), along with other clock controlled genes (inset in Figure 1). PER/CRY protein complexes accumulate in the cytoplasm over the day and later relocate into the nucleus where they inhibit the activity of the CLOCK–BMAL1 (or NPAS2–BMAL1) complex. This shuts down *Per*/*Cry* transcription during the night. After degradation of nuclear PER/CRY complexes toward the next morning, the inhibition of CLOCK–BMAL1 is released and a new cycle begins.

## Circadian and Stress Regulation of the HPA Axis

The HPA axis is a key in the regulation of stress responses, with glucocorticoids mediating intermediate and chronic adaptation to stressful stimuli, complementing the rapid response of catecholamines, both secreted from the adrenal gland. The rhythmic regulation of catecholamines and other adrenal hormones is discussed elsewhere (6). Rhythmic regulation of glucocorticoid release (Figure 1) allows for anticipation of daily timing of energy-demanding situations. In addition, glucocorticoid rhythms play a key role in the systemic coordination of circadian rhythms by resetting cellular clocks downstream of the SCN.

During stress, the brainstem and limbic forebrain stimulate corticotrophin-releasing hormone (CRH) and arginine vasopressin (AVP) secretion from neurosecretory neurons of the paraventricular nucleus of the hypothalamus (PVN) (7). *Via* the hypophyseal portal system, these reach anterior pituitary corticotrophs, which secrete adrenocorticotrophic hormone (ACTH). ACTH then acts at melanocortin type-2 receptors (MC2R) in the adrenal cortex to stimulate production and release of glucocorticoids. Negative feedback from glucocorticoids acts at the level of CRH in the hypothalamus and ACTH in the pituitary (8, 9). In addition, PVN CRH expression is indirectly controlled by the SCN (8–11).

Glucocorticoids bind to glucocorticoid (GR) and mineralocorticoid receptors (MR) in target tissues. Unlike the MR, which is almost constantly activated by glucocorticoids, GR – widely expressed in the brain and periphery, but not in the SCN (12) – activation occurs only during glucocorticoid peak levels (13). This means that GR activation may occur during the peak of the circadian rhythm even during the ultradian trough, but ultradian peaks during the circadian nadir may not be sufficient for activation (14, 15). GR binds to glucocorticoid response elements (*GRE* or *nGRE*) to regulate transcription of target genes (16). *GRE* are present in the promoter region of the clock genes *Per1, Per2, Npas2*, and various clock controlled genes involved in the synchronization of peripheral circadian rhythms (17).

Glucocorticoid circadian rhythms peak slightly before the onset of the active phase, which is during the night for most rodent species and during the day for humans (18). This rhythm overlays a more dynamic ultradian pattern for both ACTH and glucocorticoid secretion (19) driven by feedback between glucocorticoids and ACTH release at the pituitary (20) and intra-adrenal feedback of glucocorticoids (21). The presence of GR in the adrenal cortex (22, 23) and the demonstrated inhibitory effects of exogenous corticosterone on the ACTH-stimulated corticosteroid synthesis could potentially play a role in local regulation of glucocorticoid secretion in the adrenal gland. Circadian glucocorticoid rhythms can persist independent of the SCN (24–26). Given the influence that circadian rhythmicity of glucocorticoids may have on peripheral clock function, it is perhaps not surprising that the HPA axis, which is acutely activated in stressful situations, is unlikely to be the main driver of the circadian rhythm of these hormones. Indeed, there are several components of the HPA axis which, although they express circadian rhythmicity, do not synchronize well enough to explain downstream endocrine rhythms (3, 10, 27). The circadian influence of the SCN on the HPA axis also occurs through autonomic innervation of the adrenal gland (28, 29), with SCN-dependent rapid induction of *Per1* expression being stimulated in the adrenal gland following a light pulse (30). In line with this, splanchnic nerve transection results in dampened circadian glucocorticoid rhythm in rats (31, 32).

## Local Regulation of Glucocorticoid Rhythms

A circadian rhythm of steroid release was first demonstrated in isolated Syrian hamster adrenals [*Mesocricetus auratus*, See Ref. (33)], and rhythmic glucocorticoid concentrations persist under constant peripheral CRH infusion in *CRH* knockout mice (34), or in hamsters with natural loss of ACTH rhythm (35). Adrenal circadian rhythms can however be altered by ACTH, which stimulates *PER1* and *BMAL1* expression *ex vivo* in human tissue (36) and shifts clock rhythms in isolated adrenals of *Per2:LUC* reporter mice (37).

Circadian clocks within the adrenal gland also play a role in the rhythmic regulation of the HPA axis, both in glucocorticoid production and in sensitivity to ACTH. Robust clock gene expression rhythms have been demonstrated in the adrenal cortex of rodents and primates (38–43), and several steroidogenic genes show circadian expression (31, 40, 44). Work in transgenic mice has shown that those lacking genes of the positive arm of the TTL produce lower levels of corticosterone (45, 46), while those with mutations in the negative arm are chronical hypersecreters (47, 48). Evidence for the importance of adrenocortical clocks in regulating ACTH sensitivity comes from isolated adrenal tissue responses to ACTH, which differs across the day and is very low in mice deficient for *Per2* and *Cry1* (41) or *Bmal1* (46). This is further supported by evidence from primate adrenal explant studies, where knockdown of *Cry2* and subsequent downregulation of *Bmal1* lead to attenuated ACTH responses (49). Together, these studies suggest that the local adrenal clock is important for regulating the circadian glucocorticoid rhythm independent of systemic influences such as during stress, and may explain the high amplitude of glucocorticoid rhythm in the face of comparably low variations in ACTH concentrations.

## HPA Axis Interaction with the Immune System

**Figure 2:**
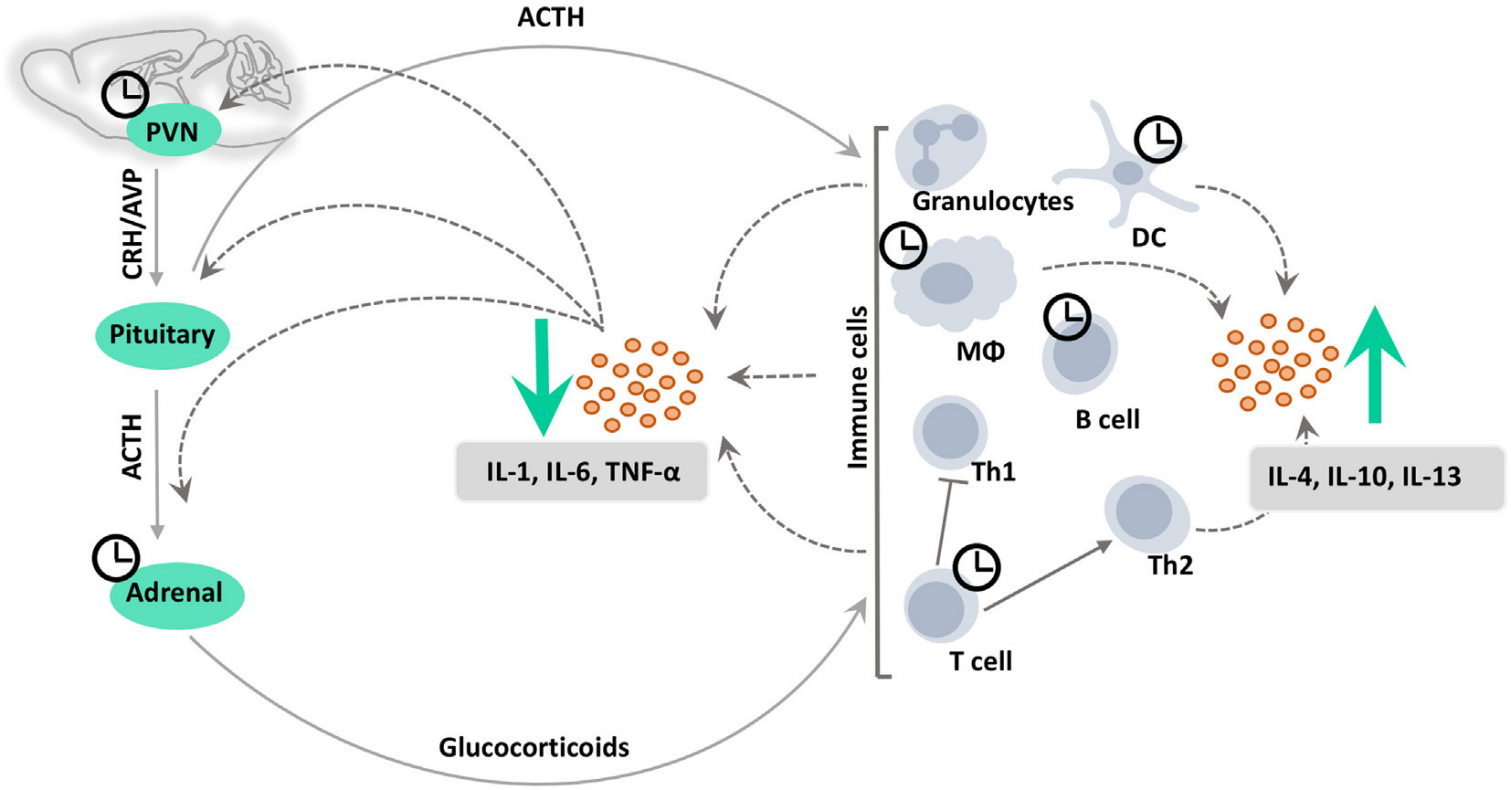
**Circadian clocks in HPA axis-immune system crosstalk**. Immune cells can activate the HPA axis *via* cytokines such as tumor necrosis factor-alpha (TNF-α) and interleukins (IL-1/6) at the level of the paraventricular nucleus (PVN) of the hypothalamus as well as at the pituitary and adrenal, stimulating the production of glucocorticoids. Glucocorticoids in turn act on the receptors on the surface or in the cytoplasm of immune cells to suppress the induction of pro-inflammatory responses, and to promote a shift from T helper cell type 1 (Th1) toward T helper cell type 2 (Th2)-mediated humoral immunity. This inhibits the production of pro-inflammatory cytokines, while promoting the production of anti-inflammatory cytokines, such as interleukin-4, interleukin-10, and interleukin-13 (IL-4/10/13) by various immune cells. In addition, ACTH exerts direct anti-inflammatory and immune-modulating effects *via* the melanocortin system. CRH, corticotropin-releasing hormone; AVP, arginine vasopressin; DC, dendritic cell; MΦ, macrophage.

A bidirectional communication exists between the HPA axis and the immune system (Figure 2). It is well understood that immune cells can activate the HPA axis *via* cytokines such as tumor necrosis factor-alpha (TNF-α), interleukins (IL-1, IL-6), and the type-I interferons (IFNs) (50–53). Interestingly, some cytokines can activate the HPA axis *via* different mechanisms. Although primarily acting on the PVN to stimulate CRH release (54–56), they also have direct action at the level of the pituitary and adrenal (57, 58).

At the same time, glucocorticoids can affect viability and function of many immune cell types, including T cells, B cells, monocytes, macrophages, and granulocytes (59, 60). Glucocorticoids suppress the synthesis and release of cytokines, thereby protecting the host organism from the detrimental consequences of a long-term hyperactivity of the immune system [reviewed in Ref. (61)]. Pioneering work by Hench, Kendall, and Reichstein demonstrated the immunosuppressive actions of glucocorticoids almost 70 years ago (62). Nevertheless, glucocorticoids are still the most widely used and most effective treatment to control allergic, autoimmune, inflammatory, and hematological disorders (63). GR are found in almost all types of immune cells, and upon activation tether and trans-represses pro-inflammatory regulators such as nuclear factor kappa-light-chain-enhancer of activated B-cells (NF-κB) and activator protein 1 (AP-1) (61, 64) by activating anti-inflammatory molecules such as glucocorticoid-induced-leucine zipper [GILZ (65)], MAPK phosphatase-1 [MKP-1 (66)], annexin-1 (67), mitogen-inducible gene-6 [Mig-6 (68)], and SRC-like adaptor protein 1 [SLAP (69)].

In contrast to the well-described immunosuppressive effects recent studies indicate that glucocorticoids can also have permissive or even stimulatory effects on immune processes [reviewed in Ref. (70–72)]. It is thought that acute stress enhances, while chronic stress suppresses the peripheral immune response (70), but the mechanism of this dual role is not well understood. Several studies report that glucocorticoids induce expression of innate immune-related genes, including members of the toll-like receptor (TLR) family, such as TLR2 and TLR4 (73–75). Glucocorticoids also rapidly induce a central component of the inflammasome, NLRP3, in macrophages, which stimulates secretion of pro-inflammatory cytokines (76).

In addition, glucocorticoids regulate adaptive immune responses by influencing cell trafficking to the sites of inflammation and by suppressing T helper cell type 1 (Th1) and enhancing T helper cell type 2 (Th2) cytokine-driven responses (77, 78). Consequently, in contrast to the traditional view of glucocorticoids as generally immunosuppressive hormones, glucocorticoids are now more accurately regarded as immune modulators.

As an alternative to the glucocorticoid treatment of chronic inflammatory diseases such as multiple sclerosis, the use of ACTH has recently been re-employed, appearing to act not only indirectly by stimulating glucocorticoid production but also by a direct anti-inflammatory effect *via* the melanocortin system [reviewed in Ref. (79–81)]. Melanocortin receptors (MCRs) are found on lymphocytes and macrophages (82–84). Anti-inflammatory effects of ACTH are mediated primarily by MC1R and MC3R, while immune-regulatory effects rely on MC5R (80). Such glucocorticoid-independent effects of ACTH have been demonstrated after lipo-polysaccharide (LPS)-stimulated production of IL-1ß and TNF-α in human blood samples (85), in a rat gout model (86), and in TNF-α-induced acute kidney disease in rats (87). Furthermore, ACTH can reduce neutrophil infiltration *via* MC3R (88).

## Circadian Interaction of the HPA Axis with Immune Function

In mammals, the circadian clock is an important regulator of the immune system, allowing the organism to anticipate daily changes in activity and the associated risk of antigen encounter. Circadian rhythms are found in multiple aspects of immune function, such as recruitment of immune cells to tissues, antigen presentation, lymphocyte proliferation, TLR function, and cytokine gene expression (89, 90). Furthermore, several inflammatory diseases, such as bronchial asthma and rheumatoid arthritis vary in severity over the course of the day, implicating a circadian regulation of vulnerability (91, 92). Animal studies have revealed that circadian rhythm disruption by shift work or chronic jet lag leads to a dysregulation of the immune system and a higher risk for several pathologies (93, 94).

Molecular clocks have been characterized in various immune cells, including macrophages, dendritic cells, and T and B lymphocytes (95–97). In humans, under constant routine conditions, administration of exogenous glucocorticoids 10 h after awakening can entrain circadian rhythms in peripheral blood mononuclear cells (PBMCs) without changing plasma melatonin and cortisol rhythms, thus linking HPA axis regulation to immune cell function. In line with this, oral administration of synthetic hydrocortisone shifts the expression of *BMAL1* and *PER2/3* in PBMCs by 9.5–11.5 h (98).

Stress-induced alterations of HPA axis rhythmicity can lead to wide-spread alterations in innate and adaptive immune responses and contribute to the development and progression of some types of cancer in animals (99, 100). In humans, data are less clear, with a positive association between stress and breast cancer observed in some (101), but not in other studies (102). In another context, stress-induced inflammatory priming of microglia was influenced by time of day in rats. Animals exposed to stress during the rest phase showed enhanced neuroinflammatory responses to an LPS challenge compared with animals experiencing stress during the active phase (103). Whether these effects involve circadian alterations remains to be shown. Of interest in this context, pulmonary antibacterial responses in mice appear to be gated by circadian clocks residing in the epithelial club cells lining the pulmonary airways, entrained by glucocorticoids (104).

Not only does the circadian clock regulate the immune system but immune status also feeds back on circadian rhythms. For example, administration of LPS resets activity rhythms in mice (105), and transiently suppresses *Per2* and *Dbp* expression in the SCN and liver of rats (106). LPS treatment increases AVP release from SCN explants (107), and TNF-α treatment downregulates SCN *Dbp* expression and causes prolonged rest periods during the active phase in rodents (108).

Recent studies reveal blunted circadian cortisol rhythms and attenuated stress responses in patients with allergic diseases, such as bronchial asthma, allergic rhinitis, atopic dermatitis, and extensive nasal polyposis (109–112). In patients with sepsis hypercortisolism persists despite low ACTH levels, suggesting that non-ACTH-mediated mechanisms are involved in the maintenance of high glucocorticoid levels. Interestingly, circadian rhythm of both ACTH and cortisol secretion were shown to be blunted in these patients (113). Furthermore, neonatal endotoxin exposure reprograms HPA axis development in rats, leading to ACTH and corticosterone hyper-responsiveness later in life (114).

## Conclusion

Cellular circadian clocks in central and peripheral tissues interact to regulate the activity of the main endocrine axes. Our improved understanding about the systemic regulation of HPA axis circadian rhythms and the interplay of clock and stress functions in this context may help to optimize current treatment strategies for many immunological disorders. For example, in chronic diseases such as rheumatoid arthritis, timed administration of exogenous glucocorticoids at specific times of day may improve therapeutic effectiveness and reduce negative side effects, since lower doses are required (115). At the same time, the importance of glucocorticoids in the coordination of the circadian timing system itself has so far been largely neglected in clinical settings. Stabilizing circadian HPA axis regulation may protect against adverse external influences such as stress and infection, thus protecting the body against some of the most frequent threats of the 24/7 globalized society.

## Author Contributions

RD, OM, and HO discussed the concept, compiled the literature, and wrote the paper.

## Conflict of Interest Statement

The authors declare that the research was conducted in the absence of any commercial or financial relationships that could be construed as a potential conflict of interest.
